# Supplementary material for: Learning strategies and their correlation with academic success in biology and physiology examinations during the preclinical years of medical school
Source: PLoS One. 2021 Jan 22;16(1):e0245851. doi: 10.1371/journal.pone.0245851 (PMC7822504; doi:10.1371/journal.pone.0245851)
Supplement: Attachment — Submitted filename: Pone-D-20-27667 Review.pdf [file pone.0245851.s001.pdf]

This study looks at different study strategies adopted by preclinical medical students at a German university and investigates whether these choices impact the academic performance for two basic science topics, biology and physiology. The authors also analyze whether students change their strategy from the first to the second year of medical school. The authors mention that they see learning success (examination scores) differences between groups of students with different learning strategies, but none of these differences is statistically significant. That may be due to the small sample size. The second question about students changing their learning strategy would also be of interest, but again it is hampered by the small sample size. These drawbacks of the study make it very preliminary and we can't draw any useful conclusions at this time. I would advise the authors to collect more data and if no significant learning success differences between learning strategies become apparent, to concentrate on the second question. It would be of interest to evaluate why students change their learning strategy and whether that makes a positive, negative or no difference for their academic success of these students.

Nice short title that describes that topic and the goal of the study. I would advise to reword the title and to avoid the word "predictor". What is the authors look at is a correlation (and they do not find any).

The introduction is well organized, concise and develops the problems addressed in this project.

The English of the manuscript, specifically the abstract, could be improved. Some of the word choices, although not wrong, are suboptimal. See a few examples below.
For example: "We here explored the learning strategies applied during the preclinical years, whether they are constant traits or subject to change and how they impact on the academic success."
Better: "In the project described in this manuscript we explored the learning strategies used by medical students at our university during their preclinical years. We investigated whether these learning strategies were constant or changed over time and whether they correlated with academic success."

The abstract misrepresents the finding of the study. As academic differences between different study strategies are not statistically significant, we can't draw any conclusion as to which strategy/ies is/are best. The conclusion section is not based on the data, but are simple truisms that are independent of the results presented in the described work.

The Methods section is missing a lot of important information.
I would appreciate more background about the structure of the German preclinical curriculum as it is used at the University of Rostock. Clearly describe the situation, like the size of the entire class (224) and its composition? Were the study participants a representative sample of the class? Which students were eliminated from the study and for what reasons. How and when were the surveys offered? There is only a very superficial description of how the learning outcome was measured. What is the role of the self-assessment? It appears that only results of

a small biology/physiology examinations were used, leaving open that other learning strategies might be a better fit for other topics (anatomy, biochemistry, pharmacology etc.). It would improve the analysis if additional or more general examination scores would be used for the correlation with the different learning strategies used by different students.
The statistical analysis appears to be appropriate.

Another significant problem of the study is that the academic success was measured using different examination topics (as this is a longitudinal study, there is no way to change this aspect), biology for year 1 and physiology for year 2. At least this needs to be critically discussed.
Also, it is never clearly stated that both topics show no statistically significant differences for the different learning strategies. That may be a direct result of the small sample size. You can't discuss the differences in the result section if they are not statistically significant.
The most interesting result is that many students keep their initial study strategy, although some change. Again, the small sample size does not allow a more detailed analysis why these changes occur and how they impacted individual student's performance.

Good that the authors included a paragraph about the limitations of their study. I would add a title "Limitation of the study" and organize this paragraph as a subsection of the discussion.

Limitations are that this analysis looks at preclinical learning, not on the students clinical abilities. There is nothing wrong with that, however, this limitation needs to be stated. As the examinations only cover biology/physiology, do NOT use the general term "academic success", but make clear that it is only academic success in these subjects. Without further information and analysis, academic success can't be assumed for other preclinical and clinical subjects.

Smaller issues:
Define acronyms at their first appearance and independently in the abstract. E.g., LIST is never defined.
"Medical school" should be "medical school".
"Examinations", not "exams".
"Likert", not "LIKERT". Likert is a name (of the social psychologist Rensis Likert), not an acronym.
Not "MC-questions", but the correct acronym is MCQ for Multiple Choice Question.
"…and sat the biology exam." That is not a complete sentence.
