# Supplementary material for: Learning strategies and their correlation with academic success in biology and physiology examinations during the preclinical years of medical school
Source: PLoS One. 2021 Jan 22;16(1):e0245851. doi: 10.1371/journal.pone.0245851 (PMC7822504; doi:10.1371/journal.pone.0245851)
Supplement: Attachment — Submitted filename: rebuttal letter.docx [file pone.0245851.s003.docx]

PONE-D-20-27667R1
Learning strategies and their correlation with academic success in biology and physiology examinations during the preclinical years of medical school
PLOS ONE

Dear Reviewers,

A Happy New Year to you – and thank you for your friendly comments. Please, find below our responses:

Reviewer #1: The authors have addressed all my previous concerns. The only remaining issue is the order of the sections. The Methods section should go before results, and not at the end of the manuscript.

Done!

Reviewer #2: I am very pleased with the corrections, clarifications and additions the authors have included in their revised manuscript. Their conclusions and the presentation are much clearer now. I think they understand that the small sample size and the limited number of topics reduce the impact of their study.
I have one remaining request, the real p-values need to be presented in Tables 1 and 2 and also for Figure 2. Just giving p-value ranges by number of stars is not sufficient.

Exact p-values have been reintroduced. Replacements by asterisks were introduced into the former version in order to accommodate a comment by Reviewer #1. We therefore feel a little bit between rock and had place…..

Thanks to both of you for your time and effort!
